# Markers of Alzheimer's Disease in Primary Visual Cortex in Normal Aging in Mice

**DOI:** 10.1155/2017/3706018

**Published:** 2017-09-12

**Authors:** Luis Fernando Hernández-Zimbrón, Montserrat Perez-Hernández, Abigail Torres-Romero, Elisa Gorostieta-Salas, Roberto Gonzalez-Salinas, Rosario Gulias-Cañizo, Hugo Quiroz-Mercado, Edgar Zenteno

**Affiliations:** ^1^Research Department, Asociación para Evitar la Ceguera en México, “Hospital Dr. Luis Sanchez Bulnes” IAP, 04030 México City, Mexico; ^2^Divisón de Ciencias Biológicas de la Salud, Universidad Autónoma Metropolitana, Unidad Iztapalapa, Ciudad de México, Mexico; ^3^Neuroscience Division, Institute of Cellular Physiology, UNAM, Ciudad Universitaria, Ciudad de México, Mexico; ^4^Cell Biology Department, Centro de Investigación y de Estudios Avanzados del IPN, Ciudad de México, Mexico; ^5^Department of Biochemistry, School of Medicine, UNAM, Ciudad Universitaria, México City, Mexico

## Abstract

Aging is the principal risk factor for the development of Alzheimer's disease (AD). The hallmarks of AD are accumulation of the amyloid-*β* peptide 1–42 (A*β*42) and abnormal hyperphosphorylation of Tau (p-Tau) protein in different areas of the brain and, more recently reported, in the visual cortex. Recently, A*β*42 peptide overproduction has been involved in visual loss. Similar to AD, in normal aging, there is a significant amyloid deposition related to the overactivation of the aforementioned mechanisms. However, the mechanisms associated with visual loss secondary to age-induced visual cortex affectation are not completely understood. Young and aged mice were used as model to analyze the presence of A*β*42, p-Tau, glial-acidic fibrillary protein (GFAP), and presenilin-2, one of the main enzymes involved in A*β*42 production. Our results show a significant increase of A*β*42 deposition in aged mice in the following cells and/or tissues: endothelial cells and blood vessels and neurons of the visual cortex; they also show an increase of the expression of GFAP and presenilin-2 in this region. These results provide a comprehensive framework for the role of A*β*42 in visual loss due to inflammation present with aging and offer some clues for fruitful avenues for the study of healthy aging.

## 1. Introduction

Traditionally, the aging population has been associated with developed countries, but currently two-thirds of the world's oldest persons live in developing countries, where the elderly population is growing faster than in developed regions.

AD is the most common cause of dementia in older people, and it is estimated that 27 million people are affected worldwide [1, 2]. As the life expectancy of the population increases, the number of affected individuals is predicted to present a threefold increase by 2050 [2, 3]. Different risk factors have been associated with the development of AD, such as environmental, dietary, and pathological factors, altered glucose metabolism, chronic inflammation, gender, and oxidative stress. Nevertheless, age continues to be the main risk factor for AD, although early-onset disease can occur before the age of 60 years [4, 5].

AD is a paradigm of a neurodegenerative disorder that is caused by the detrimental progression of age-dependent loss of cognitive function. The hallmarks of this disease are accumulation of amyloid aggregates (also known as amyloid plaques), principally constituted by abnormal local deposits of A*β*42 in the extracellular brain parenchyma and the hippocampus, as well as the formation of neurofibrillary tangles within the neurons in the aforementioned regions [6]. These neurofibrillary tangles consist of cross-linked protein strands that generate a double helix structure; the principal component of these tangles is the pathologically hyperphosphorylated Tau protein [7].

The production of A*β*, a critical event in AD, results from the cleavage of the amyloid precursor protein (APP), whose levels are high in AD. This peptide is toxic and induces several detrimental effects on cells, like cell membrane disruption, excessive production of reactive oxygen species, interactions with several proteins that affect their normal function, synaptic failure, chronic local inflammation, glial hyperactivity, and cell death [8]. These changes have been studied and identified mainly in brain areas such as entorhinal, prefrontal, and visual cortices, hippocampus, and olfactory bulb.

In addition, it has been reported that AD patients lose certain visual functions that are not correlated to structural damage of the eye but with loss of neurological function. A*β*42 peptide toxicity has been related to several disrupted molecular mechanisms in normal vision; for example, inhibition of long-term potentiation and cognitive processes [9, 10] increases the proinflammatory effects in the occipital visual cortex and induces gliosis and apoptosis among other toxic effects. These alterations have been correlated with ophthalmic disorders such as age-related macular degeneration (AMD) and glaucoma [11–13]. However, there is not enough information about these pathological changes in the visual cortex during normal aging.

Aging is accompanied by chronic inflammation, demonstrated by the increase of inflammatory mediators such as cytokines and oxidative stress markers and chronic antigenic stress and influenced by the genetic background [14–16]. Chronic inflammation appears to be involved in the pathogenesis of some age-related diseases such as AD, atherosclerosis, diabetes, age-related macular degeneration, and cancer [17, 18]. As in Alzheimer's disease, aging is also characterized by the accumulation of A*β*42 in “inflammaging,” a term used to highlight the importance of inflammation in many age-associated diseases [19]; as previously mentioned, the presence of A*β*42 is associated with vision loss. The presence of multiple A*β*42 reservoirs in the eye (especially in the retina and the optic nerve) induces different pathologies that lead to potentially blinding disorders [20]. However, the presence of A*β*42 in the visual cortex and the role it plays in vision loss, related to normal aging, have not been described.

Brains of 4-month-old and 25-month-old C57BL/6J mice were used as an aging model. Visual cortices (VC) were analyzed to evaluate whether an AD-like pathology develops during normal aging in this area.

Our results demonstrated intracellular accumulation of A*β*42, A*β*42 deposition in blood vessels, and disturbances in the pattern of p-Tau protein distribution in the VC of 25-month-old mice. This murine model also showed overexpression of the enzymes involved in the production of A*β*42 and an increase in the number of astrocytes expressing GFAP protein in the aged mice compared to the young ones.

## 2. Materials and Methods

### 2.1. Animal and Animal Care

Young (4-month-old and 25-month-old) male C57BL/6J mice were maintained on a 12-hour light/dark cycle in a temperature-controlled room, within a clean air box, and food was provided ad libitum (NutriCubo, Purina, USA). The animals were maintained and treated in accordance with the NORMA Oficial Mexicana NOM-036-SSA2-2002, the National Institutes of Health Guidelines for Animal Treatment, and the Ethics Committee of the Asociación para Evitar la Ceguera en México, “Hospital Dr. Luis Sanchez Bulnes” IAP.

### 2.2. General Procedure

Mice were randomly separated into two experimental groups (*n* = 6 per group). Group 1 was composed of 4-month-old animals and group 2 was composed of 25-month-old mice.

The visual cortices of control and aged mice were obtained for IMHQ assays. Briefly, six animals from each group were transcardially perfused with 4% paraformaldehyde (Sigma-Aldrich Chemie, Germany) in 0.1 M phosphate buffer (J.T. Baker, NJ; PB, Tecsiquim; pH 7.4) for the immunohistochemistry assays. Male 4-month-old and 25-month-old C57BL/6J mice were evaluated in the study. Animals were perfused transcardially with phosphate-buffered saline (PBS) and 4% (w/v) paraformaldehyde under sedation. The brains were postfixed in 4% paraformaldehyde for 20 h and immersed in a 30% sucrose solution (w/v) in PBS for 24 h.

Coronal sections (20 *μ*m) from visual cortex were cut on a freezing microtome (Leica CM3050s) and mounted serially. Slides were used for immunofluorescence detection.

### 2.3. Immunohistochemistry and Immunofluorescence

Rabbit monoclonal anti-A*β*42 antibody (obtained from Abcam, MA, USA) was used to detect the A*β*42 peptide. Goat polyclonal anti-GFAP and rabbit polyclonal anti-p-Tau and rabbit anti-presenilin-2 (PS2) antibodies were from Santa Cruz Biotechnology (CA, USA). Alexa Fluor 594 goat anti-rabbit IgG (H+L), Alexa Fluor 488 mouse anti-goat IgG (H+L), Alexa Fluor 488 goat anti-rabbit IgG (H+L), and Alexa Fluor 594 mouse anti-goat IgG (H+L) were from Molecular Probes, OR, USA.

For the double immunofluorescence (IF) assays, rabbit monoclonal anti-A*β*42/goat polyclonal anti-p-Tau (dilution 1 : 100) antibodies were used and visualized with Alexa Fluor 594 goat anti-rabbit IgG (H+L) and Alexa Fluor 488 mouse anti-goat IgG (H+L). Besides, rabbit monoclonal anti-PS2/goat polyclonal anti-p-Tau (dilution 1 : 100) antibodies were used and visualized with Alexa Fluor 488 goat anti-rabbit IgG (H+L) and Alexa Fluor 594 mouse anti-goat IgG (H+L). Samples were mounted onto glass slides in VECTASHIELD Medium (Vector Laboratories, Burlingame, CA, USA) containing DAPI. Representative brain sections from each group were processed in parallel next; these sections were examined with an Olympus BX41 Microscope (Japan) and photographed with an Evolution-QImaging Digital Camera Kit (Media Cybernetics, Rockville, MD, USA) for DAB reaction and the double IF assays were observed through a Leica DM-LS epifluorescence microscope at 40x and 100x (Leica Microsystems, Wetzlar, GmbH, Germany). The fluorochromes were visualized with their specific filters and analyzed in three channels.

#### 2.3.1. Image Analysis

Fluorescence pixel intensities were measured in several regions of interest (ROIs) within each image using ImageJ. Average pixel intensities were calculated from five ROIs for measurements in different regions from each image. The measures were realized in six animals per time point. All signal intensities were background-subtracted from the average of three.

#### 2.3.2. Statistical Analysis

All of the data are expressed as mean Chi-square for trends and Fisher's exact tests were used for multiple comparisons. Prism GraphPad software was used (Systat Software, Inc., Point Richmond, CA, USA).

## 3. Results

**Figure 1:**
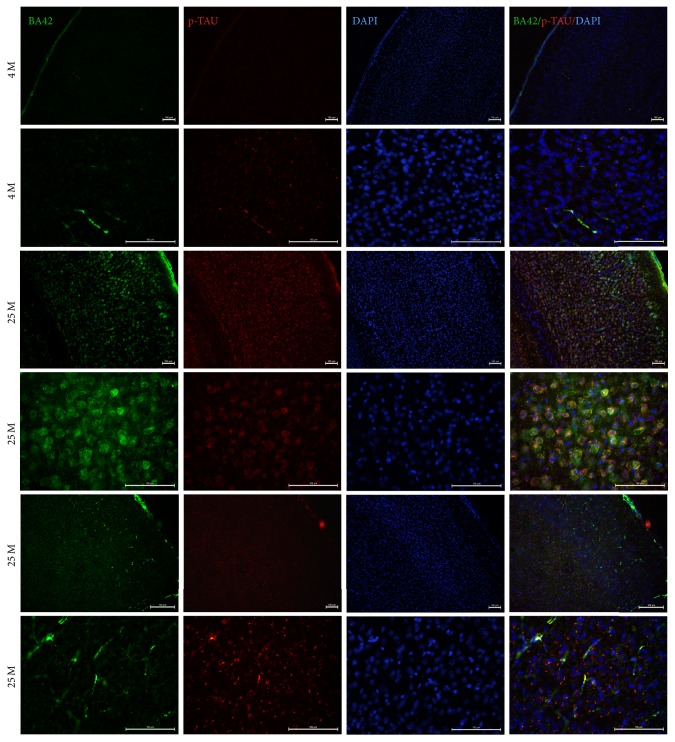
Double IF to detect A*β*42 (green channel) and p-Tau (red channel) in VC of 4 M and 25 M old mice. 15 um thick brain tissue sections of VC from mice were used. DAPI stain for nuclei (DAPI) and Merge are shown. Observe the intracellular accumulation, blood vessel deposition of A*β*42 peptide, and the increase of p-Tau signal in VC from 25 M old mice. Some tissues of mice show more A*β*42 deposition in blood vessels (two bottom last lines). Scale bar: 100 *µ*m.

**Figure 2:**
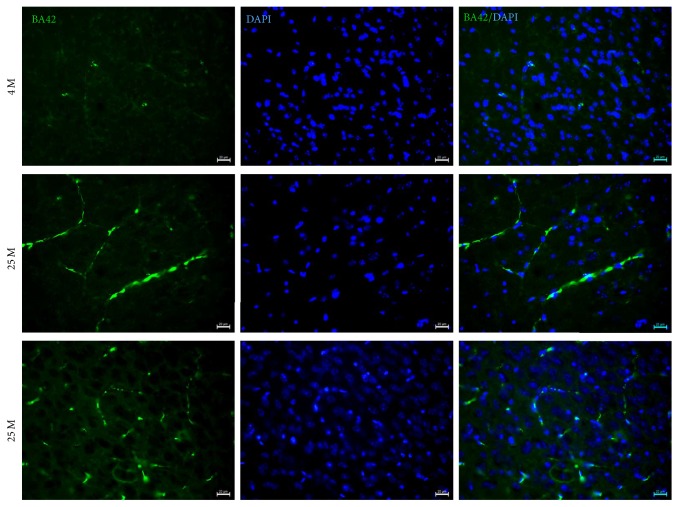
Double IF to detect A*β*42 in the VC of 4 M and 25 M old mice. 15 um thick brain tissue sections of VC from mice were used. Observe A*β*42 accumulation in blood vessels in the VC of 25 M old mice. Scale bar: 20 *µ*m.

**Figure 3:**
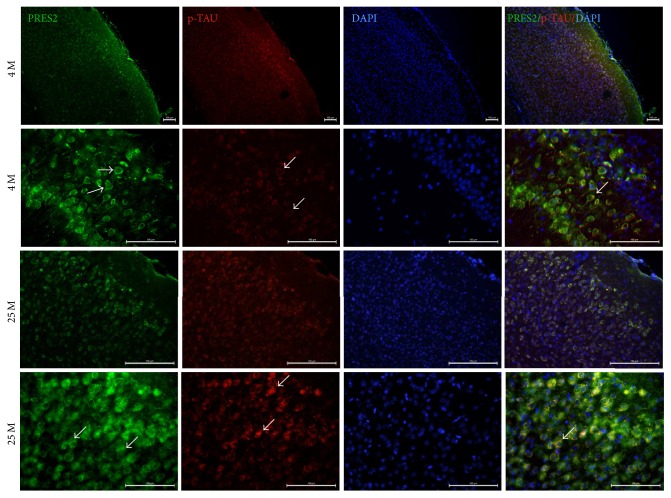
Double IF assays to evaluate the overexpression and changes in the localization of presenilin-2. Observe the increase of PS2 expression and the change in the localization of this enzyme in aged mice (25 M, white arrows). In young mice, PS2 presents a cytoplasmic expression pattern, but, in aged mice (25 M), it is apparently located in the cell membrane (white arrows). Scale bar: 100 *µ*m.

We studied the deposition of A*β*42, changes in Tau expression patterns, GFAP overexpression, and the enzymes involved in the production of the A*β*42 peptide in the visual cortex of 4-month-old (4 M) and 25-month-old (25 M) aged symptomatic mice (C57BL/6J WT). To evaluate the overproduction and accumulation of the A*β*42 peptide in the VC, we performed double immunofluorescence assays in brain sections derived from control (4 M) and aged mice (25 M). The double IF assays showed qualitative increases in the intracellular accumulation and blood vessel deposition of A*β*42 (Figures 1 and 2) and an increase in its expression, as well as a change in p-Tau's distribution in the VC (Figures 1, 2, and 3) of 25 M aged mice.

In order to confirm A*β*42 deposition in blood vessels, we repeated the IF to detect A*β*42 accumulation in 4 M and 25 M aged mice. Again, there is an increase of A*β*42 in blood vessels of 25 M old mice (Figure 2).

The A*β*42 peptide is a product of the proteolytic cleavage of amyloid precursor protein (APP). APP's cleavage is done first by beta-secretase, followed by a second cleavage by the gamma-secretase complex. This complex is a multisubunit protease comprised of four components: presenilin-1 and presenilin-2 (PS1 and PS2, resp.), nicastrin, anterior pharynx defective-1 (APH-1), and presenilin enhancer 2 (PEN-2). Among these proteins, PS2 is a transmembranal protein and it has been confirmed as the main enzyme involved in the production of A*β*42 in AD. To evaluate changes in PS2 expression which could be related to the increase in the production of A*β*42, we performed IF assays in the same aging model. In these assays, because PS2 is a membranal protein (as mentioned before), we decided to use p-Tau to mark microtubules to identify the localization of PS2.

As shown in Figure 3, there is an increase of PS2 expression and there are changes in the expression pattern of this enzyme in the VC of aged mice compared to the VC of young mice. Interestingly, we observed changes in the localization of p-Tau protein in aged mice (white arrows in Figure 3).

As shown in Figure 3, there is an increase of PS2 expression and there are changes in the expression pattern of this enzyme in the VC of aged mice compared to the VC of young mice. Interestingly, we observed changes in the localization of p-Tau protein in aged mice (white arrows in Figure 3).

**Figure 4:**
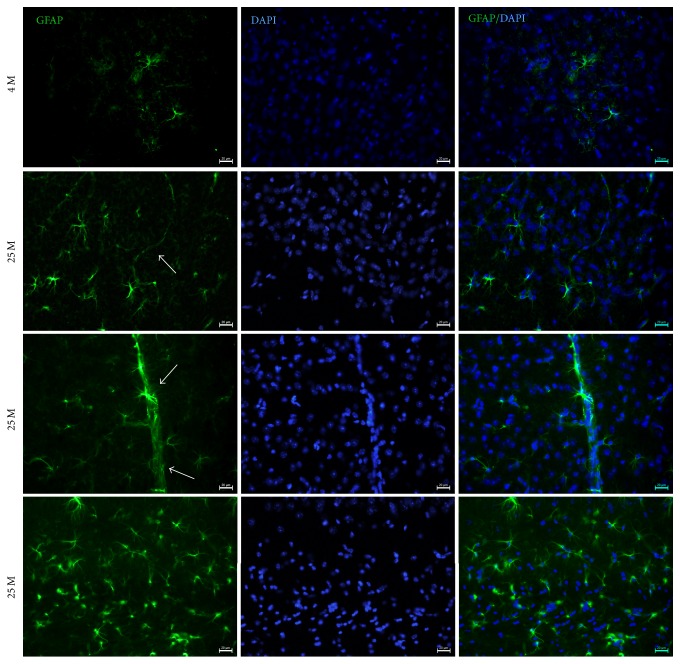
Double IF to detect glial fibrillary acidic protein (GFAP), an inflammation marker (green channel), in the VC of 4 M and 25 M old mice. 15 um thick brain tissue sections of VC from mice were used. DAPI stain for nuclei (DAPI) and Merge are shown. Observe GFAP overexpression and an increased number of astrocytes in the VC of 25 M old mice. Scale bar: 20 *µ*m.

To evaluate whether A*β*42 deposition induces an inflammatory response in aged mice, we performed IF assays to detect astrocytes using a GFAP antibody. GFAP is a commonly used marker for astrocytes, and it has been related to brain inflammation and to the proper functioning of the blood-brain barrier in health. In this aging model, we observed an increase in the number of astrocytes positive to GFAP in 25 M mice (Figure 4). Besides, there were large numbers of astrocytes associated with blood vessels in aged mice.

Finally, to demonstrate these results in a semiquantitative manner, we performed fluorescence intensity quantification on every protein or peptide studied in this report.

**Figure 5:**
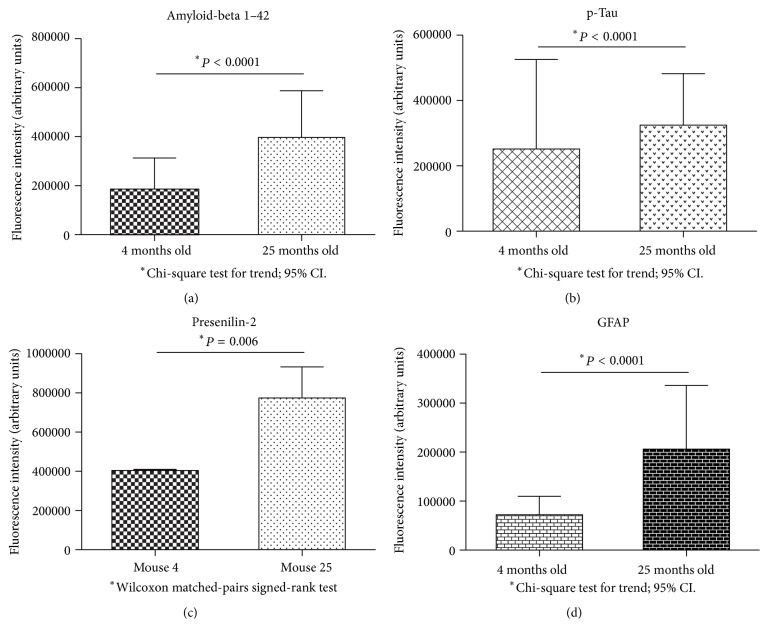
Fluorescence intensities quantification of A*β*42 peptide (a), p-Tau (b), presenilin-2 (c), and GFAP (d) on visual cortex of 4-month-old and 25-month-old mice. For all cases, data were significant. Data are in arbitrary units of fluorescence intensity; *n* = 6 animals per group.

As shown in Figure 5, we observed a statistically significant increase in the intracellular A*β*42 peptide (a), p-Tau (b), PS2 (c), and GFAP (d) in the VC of aged mice.

## 4. Discussion

In the present study, we demonstrated that, during normal aging, in mice, there is an increase of AD-like pathological changes, like intracellular accumulation and blood vessel deposition of A*β*42 in the VC; these increases were correlated with significant overexpression of one member of the gamma-secretase complex (PS2). Besides, GFAP, a common marker for astrocytes, showed an increase in its expression, and the number of GFAP-positive astrocytes also increased, indicating the activation of immunological responses in aged brains.

AD is a neurodegenerative disease with a complex and progressive pathological phenotype that is initially characterized by hypometabolism and impaired synaptic function and, subsequently, by pathological burden [21]. The A*β*42 peptide is the pathological hallmark of AD produced by the sequential cleavage of APP by *β*-secretase and the gamma-secretase complex. In contrast, the activation of *∂*-secretase leads to nonamyloidogenic processing of APP and the generation of truncated nontoxic sAPPa fragments [5, 22, 23]. Neurofibrillary tangles formed by the pathological hyperphosphorylation of Tau protein, an associated-microtubule protein that helps to stabilize microtubules, are the other AD hallmark.

The results presented herein show similar AD pathologic changes and, importantly, this model lacks other factors that could be inducing this pathology. It is important to mention that the amino acid sequence of the A*β*42 peptide in mice does not form amyloid plaques. However, our results indicate an increase of intracellular A*β*42 in the VC of aged mice which could be related to the increased expression of PS2, the main enzyme associated with A*β*42 production in AD [24, 25], and supported by previous studies that have shown that all the components of the gamma-secretase complex increase under stressful conditions, as in AD [26].

It has been reported that A*β*42 can bind to a great number of proteins and to extracellular and intracellular macromolecules that affect normal neuronal function due to increases in the production of hydrogen peroxide, induction of oxidative stress, disturbances in Ca^2+^ homeostasis, and mitochondrial dysfunction (promoting the opening of the membrane permeability transition (MPT) pores or disruption of neuronal signal transduction pathways in AD [13, 19, 27–30]); however, it is not known whether A*β*42 accumulation in the VC activates these pathological processes and, as a consequence, they are affecting normal vision. Our results showed an increase of p-Tau, another hallmark of AD. It has been demonstrated that an abnormal increase in p-Tau affects the normal functioning of microtubules, impairs intracellular communication, and, finally, induces cell death [31, 32]. As we observed in Figures 1 and 2, p-Tau not only increases its expression but also shows a change of its distribution and localization, suggesting that there may be changes in its intracellular transport in aged mice. More experiments need to be performed to evaluate the impact of this differential expression and localization of p-Tau in the VC during aging.

AD represents a chronic inflammatory state caused mainly by the presence of the A*β*42 peptide. It has been broadly reported that there is an overreactivity of immune cells, such as astrocytes and microglia, during normal aging and AD [33]. Our results show that some GFAP-positive astrocytes are associated mainly with blood vessels in aged mice, and quantification of GFAP reveals that glial response is also prominent in the VC, as observed in AD. This could be relevant, because disruption of the blood-brain barrier in AD and activation of proinflammatory mechanisms (like oxidative stress) due to the presence of A*β*42 have been previously reported. Interestingly, the A*β*42 peptide accumulates in blood vessels in 25 M old mice. This could suggest that these factors are related, and the presence of growing numbers of astrocytes in blood vessels is a protective response to avoid or repair blood-brain barrier damage caused by the A*β*42 peptide's presence in the aged visual cortex.

There are several inflammatory factors in the aging brain which originate from microglia and astrocytes, as they adopt a senescence-associated secretory phenotype [19, 34]. Some aging astrocytes release more cytokines, which is consistent with the aforementioned phenotype [35]. However, more studies are needed to demonstrate the production levels of proinflammatory cytokines in the visual cortex of aged mice. Together, these events contribute to neuronal dysfunction in primary visual areas, supposedly protected from beta-amyloid deposition [36].

In another way, in order to discuss the translation of our findings and their application in human, it is necessary to mention some interesting points about the different animal models used in aging and AD.

The simplest and best model of aging is an old organism. Mouse is an attractive model for studying mammalian biology due to the genetic manageability of its genome, ease of breeding, and the large amount of available baseline phenotypes; they are relatively economical to maintain for long-term aging studies and, more importantly, they are similar to humans genetically and physiologically [37, 38]. These similarities and differences between mouse and man in relation to studies on aging have been extensively reviewed [15, 39–42].

To replicate the pathology of AD in humans, several animal models of AD pathology have been developed. These mouse models have been useful to study the mechanisms involved in the progression of AD and to predict outcomes from pharmacological interventions. No animal model fully replicates the pathogenesis and the cognitive deficits observed in human AD and therefore it is important to understand both the utility and limitations of particular animal models.

According to the recent NIA-AA sponsored consensus reports on three defined stages in a clinical continuum for AD including preclinical and mild cognitive impairment and dementia, the latter is related to the presence and extent of neuropathological changes of AD patients observed at autopsy [43, 44]; we present here these neuropathological changes in healthy aging in mice. Our results suggest that these changes could be affecting the normal vision in AD patients as it has been previously reported.

Although the mouse might not be the perfect model for studying aging, its use as a model of mammalian biology will contribute to gaining important insights into the pathobiology of different diseases, fundamental processes involved in aging, and the relationship between aging and AD. Besides, the validation of these models is totally necessary for a better understanding of the effects of healthy aging and AD on vision loss to translate these advances to humans.

## 5. Conclusions

Our results show a significant deposition of A*β*42 peptide and overexpression of other AD markers such as p-TAU and GFAP in the VC of WT 25-month-old mice.

We suggest that the overexpression of presenilin-2 observed in our experiments may be one of the first mechanisms involved in beta-amyloid overproduction in our model and that the mechanisms related to neuronal degeneration downstream of A*β*42 accumulation could include membrane-associated oxidative stress, altered Ca2+ homeostasis, altered energy metabolism, and activation of apoptosis. These findings suggest roles for an alteration of immune responses in the aging process.

Our results demonstrate that aging is possibly related to the activation of the amyloidogenic pathway, which induces A*β*42 overproduction and intracellular accumulation in visual cortex cells; furthermore, we suggest that A*β*42 accumulation affects several important mechanisms to initialize neurodegenerative processes, such as those occurring in AD, and could be related to vision loss.

Our results aid to understand the correlation between aging and the development of neurodegenerative diseases such as AD, but additional studies are needed to further investigate the effect of the A*β*42 peptide on the VC and the retina.
